# People Living in Places with Limited Illuminance Declare Better Health and Higher Quality of Life in Environmental and Physical Domains

**DOI:** 10.3390/clockssleep8010003

**Published:** 2026-01-05

**Authors:** Jolanta Malinowska-Borowska, Anna Czupryna, Marta Buczkowska, Aleksandra Kulik

**Affiliations:** 1 Department of Chronic Diseases and Civilization-Related Hazards, Faculty of Public Health in Bytom, Medical University of Silesia in Katowice, 18 Piekarska Street, 41-902 Bytom, Poland; 2 Scientific Circle at Department of Chronic Diseases and Civilization-Related Hazards, Faculty of Public Health in Bytom, Medical University of Silesia in Katowice, 18 Piekarska Street, 41-902 Bytom, Poland

**Keywords:** light pollution, artificial light at night, Dark Sky Park, quality of life, WHOQOL-BREF

## Abstract

Background. Exposure to artificial light at night can lead to circadian disruption and health risks. It can cause mood swings, confusion, and depression. The aim of this cross-sectional study was to assess the relationship between the illuminance of urban lighting and the health of residents. Methods: This study was carried out among residents of two similar towns, one with typical street lighting and a Dark Sky Park characterized by reduced lighting. A total of 272 respondents participated in this study. A self-administered questionnaire and the WHOQOL-BREF were used among the respondents. Results. People living in the Dark Sky Park were more likely to be satisfied with their sleep (*p* < 0.001). In fact, 58.7% of Dark Sky Park residents reported no sleep problems. In the control town, only 49.25% did (*p* = 0.04). The sleep duration was similar in the two towns, but Dark Sky Park residents were statistically less likely to use sleeping pills and window blinds. People exposed to typical street lighting at night reported suffering from eye diseases, cardiovascular diseases, and mood changes more often than those living in the Dark Sky Park. The environmental and physical quality of life, as measured by the WHOQOL-BREF, were significantly higher in the Dark Sky Park residents than in the control town (*p* < 0.05). Conclusions. People living in places with limited illuminance declare better health and a higher quality of life in the physical and environmental domains.

## 1. Introduction

Light is the visible part of electromagnetic radiation in the wavelength range of 380 to 780 nm. It is the most powerful stimulating factor for life on Earth, synchronizing the circadian rhythm, which affects various physiological processes, from gene expression to the sleep–wake cycle [1].

Today, more than 80% of the world’s population and almost 100% of the inhabitants of the United States of America and Europe live under skies polluted by light [2]. Even the Arctic is polluted by light [3]. Light pollution (LP) comes in many forms, including sky glow, light trespass, glare, and over-illumination [4]. Working at night, watching television, or roadway lighting shining directly into bedrooms are examples of *artificial light at night* (ALAN), which is recognized as the cause of light pollution.

LP is not a new issue. Early concern about the growth of LP dates back to the early 20th century, when the visibility of the stars decreased due to LP, and astronomers began to look for new locations for their observatories on mountains, far away from light sources [5].

However, today, LP is not just a problem for astronomers, as LP affects not only the environment. Excessive artificial lighting can lead to disruptions in behavioural and metabolic rhythms and changes in light-regulated physiological and psychological functions of humans [6].

ALAN, which alters natural, cyclical daylight and dark conditions, can lead to serious health risks [7]. It can affect everything from the timing of hormone release in the body to the length and quality of sleep [8], consequently increasing systemic inflammation [8,9]. Visible light at night can cause mood swings, confusion, irritability, and depression due to the lack or reduced production of important neurotransmitters and neurohormones [10,11].

It is believed that ALAN may be linked to the cancer risk, as scientific studies have shown that night workers are particularly vulnerable to breast and prostate cancer due to impaired melatonin production [12,13,14,15]. In 2007, the IARC (International Agency for Research in Cancer; World Health Organization) classified circadian disruption as a probable human carcinogen [16]. The US National Toxicology Program has recently concluded that “there is a moderate evidence of a causal relationship between ALAN exposure and cancer, because ALAN may act through different mechanisms of circadian disruption and its biological effects are the same as those of well-known recognised carcinogens” [17]. According to the Scientific Committee on Emerging and Newly Identified Health Risks, research into the mechanisms and consequences of exposure to artificial light in the late evening, at night, and in the early morning is urgently needed [18].

Nowadays, places with controlled and even reduced light pollution, called Dark Sky Parks, are desirable not only for astronomers. The Dark Sky Park (DSP) is an area characterized by an exceptional starry sky and natural nocturnal habitat where LP is mitigated and natural darkness is an important educational, cultural, and natural resource. A DSP is a tourist attraction, providing a good night’s sleep and rest [19]. In such places, roadway lighting must meet the standards of the International Dark-Sky Association (IDA), for example, only warm light sources are allowed [5]. Moreover, in Dark Sky Parks, the IDA recommends that luminaires must be fully shielded to emit light only downward and minimize sky glow from upward-emitted light. More and more countries, cities, and regions are working to preserve their night skies and ensure their residents have access to them. National policies support light-sensitive wildlife populations and enhance astrotourism. However, in Poland, there are only five Dark Sky Parks. There is literature on the implementation of astronomical observations in DSPs, but there are no scientific reports concerning the health of people living there.

The aim of this study was to assess the health and quality of life of people living in a DSP characterized by reduced light pollution at night. We investigated whether selected diseases were less common among the inhabitants of the Dark Sky Park. A comparison of sleep habits reported by residents of two similar villages, one of which is a DSP was carried out. The respondents’ general quality of life was also assessed via the WHOQOL-BREF questionnaire to find out if the quality of life is better in places with less light pollution.

## 2. Results

Research material was collected among the inhabitants of two similar mountain villages in Poland, one of which has been a Dark Sky Park since 1997. The study group consisted of 272 people, 98 (36.03%) men and 174 (63.97%) women. The mean age of the respondents was 46 ± 11.46 years. In the DSP, the average duration of residence was 42.98 years, and in the control town, 40.75. The vast majority lived permanently in the same place. Only five residents of the DSP and four in the control town had lived there for less than 10 years, of which only one had lived there for less than 5 years. The characteristics of the study group are presented in Table 1.

The sleep of residents of both villages was compared. People living in the Dark Sky Park most often go to sleep at 10:00 p.m. and wake up at 6:00 a.m. They sleep 7.46 h on average. The residents of the control village usually go to bed at 11 p.m. and wake up at 6 a.m. They sleep approximately 7.39 h (*p* = 0.15).

People living in the Dark Sky Park are more often satisfied with their sleep (*p* < 0.001). As many as 58.7% of the residents of the DSP confirmed that they did not have any sleeping problems. In the control town, 49.25% of the respondents did. Moreover, 13.4% of the residents of the control town reported that they experienced sleep problems more often than once a week (*p* = 0.04). In this group, up to 17.16% of respondents use sleeping pills. Inhabitants in the control town statistically more often covered their windows with blackout curtains or blinds before going to sleep. Answers of respondents concerning sleep are presented in Table 2.

In Table 3, the distribution of responses regarding the presence of a depressed mood is shown. Residents in the control town more often reported depression and anxiety (*p* = 0.01). More than 10% of people living in the DSP (10.14%) admitted that they regularly consult a specialist, such as a psychologist or psychiatrist, whereas in the control town, this number was twice as high.

In the survey, respondents were asked about their health condition and diagnosed diseases. They could choose any number of diseases they suffer from and even enter their own answer. Cancers, cardiovascular diseases, ophthalmic diseases, and mood changes were available to choose from the list in the questionnaire because they have been suggested in the literature to be associated with light pollution.

Some inhabitants of the DSP and the control town admitted that they suffer from cardiovascular diseases such as hypertension (21.7% versus 33.6%). The differences in the number of cases of cardiovascular diseases between the residents of both towns were statistically significant (*p* = 0.028).

The incidence of all diseases was higher in the control group (Table 4). However, in addition to cardiovascular diseases, statistically significant differences were also observed for eye diseases (e.g., glaucoma) (*p* = 0.008) and depression (*p* = 0.025). For each disease, a logistic regression model was developed, incorporating quantitative predictors such as age, sleep duration, and sleeping pill administration alongside qualitative factors including sex and education. No models exhibited a statistically significant predictive value; that is why no information of this kind was included in the manuscript.

Table 5 shows the results from WHOQOL-BREF questionnaire. The overall quality of life was similar in both groups, at 3.65 ± 0.8. In both the Dark Sky Park (58.70%) and the control town (54.48%), the respondents assessed their health as satisfactory. The results obtained in all domains for both villages indicate a reduced quality of life in the social domain in comparison to other areas studied. The mean value of the environmental domain achieved for the inhabitants of the DSP was significantly higher compared to the residents of the control town (*p* = 0.01). A statistically significant difference was also observed in the physical domain (*p* = 0.04).

## 3. Discussion

Excessive artificial lighting after dark has been shown to disrupt the natural rhythm of sleep and wakefulness. Numerous experimental studies have confirmed the ability of light to suppress the secretion of melatonin and affect human sleep [20,21]. Increased access to electric lighting, as well as the use of separate bedrooms and modern soft beds, has an impact on our sleep and sleep habits connected with sleep [22].

In our study, the sleep duration reported by residents of both towns was similar. However, sleep satisfaction was greater in the Dark Sky Park than in the control town. Most people living in the control town declared experiencing sleep problems once a week, which may be associated with exposure to ALAN. Excessive street lighting at night might also influence the sleep habits. Most of the residents of the control town admitted that they had to sleep with blackout curtains. Our findings are consistent with the conclusions of Ohayon MM et al. [23]. According to those authors, exposure of residents to inappropriate roadway lighting contributes to sleep disorders. In a study of more than 19,000 individuals, those living in areas with more outdoor night lighting self-reported a lower quality and quantity of sleep, as well as greater daytime sleepiness.

Lahiri et al. measured light pollution and compared the sleep quality of people living in selected rural and urban areas in West Bengal, India. Among 512 people, a poorer sleep quality was significantly more common with increased light exposure [24]. Wright and his colleagues came to the same conclusion. In their study, during the first week, the volunteers lived without changing their routines and habits in the city, while the next week they slept in the Rocky Mountains and could not use any electronic devices. The light reaching them was continuously recorded, as well as the synthesis of melatonin in saliva. The study revealed that melatonin synthesis started 2 h before sleep and stopped 50 min before waking. Artificial lighting shifts the synthesis of melatonin, causing people to fall asleep later, and worsening the quality of their sleep [25]. In our study, the administration of sleeping pills was statistically more frequent among the inhabitants of the control town as they had a poorer sleep quality, problems falling asleep, and bouts of waking up at night.

The different outdoor lighting in the two towns is also reflected in the frequency of use of blinds and blackout curtains at home. Most of the residents of the control town admitted that they had to sleep with blinds on their windows. Excessive street lighting at night influences the habits before going to bed among the respondents.

In the study by Gooley et al., 116 individuals initially had to follow a fixed 8 h sleep schedule for 2 weeks [26]. Later, the volunteers were divided into two groups. The first group was exposed to artificial lighting at bedtime with an illuminance of 200 lux. In the second group, the illuminance was less than 3 lux. The results of the study indicate that the synthesis of melatonin in the body and the duration of its action were reduced by approximately 90 min in the group exposed to excessive light. The authors also emphasize that chronic exposure to electric light in the late evening can potentially affect not only sleep but also thermoregulation, blood pressure, and glucose homeostasis [26]. Notably, the health effects of ALAN are related to the exposure conditions and characteristics of the light, not just to the amount of light [27]. The intensity of light, the proportion of blue light, and the timing and duration of the exposure has an impact on the severity of health effects [8].

In the last decade, the strong effect of blue light on melatonin suppression has been better documented, leading to a greater awareness of its importance for human health. Melatonin exerts protective effects in the central nervous system, reducing free-radical stress and inflammation [9,28]. Therefore, disturbances in melatonin suppression have been linked with stress, insomnia, depression, thermoregulation, alertness, and the heart rate. Some studies estimate that the risk of breast cancer is 30–50% greater in countries with the highest exposure to LAN than in those with the lowest exposure [29]. Research from Israel has shown a strong correlation between breast cancer rates and light pollution levels [30]. A case-referent study in Georgia also suggests positive associations between LAN exposure and the breast cancer incidence, especially among Caucasians [31]. In this work, respondents were asked about cancers in general. The number of cases reported in the control town was greater than that reported in the DSP, but the difference was not statistically significant.

In our study, a statistically significant relationship was observed between diseases such as eye diseases, mood changes, as well as cardiovascular diseases, e.g., hypertension. In a village with typical street lighting, a larger group of people admitted to having a low mood. As many as 23% of the inhabitants of the control town often or very often experienced negative feelings such as depression or anxiety. The incidence of a subjective feeling of low mood was higher in the control town. However, it should be emphasized that it cannot be concluded that a low mood was related to the artificial lighting used in both locations. The causes of negative feelings may be different and may result from factors not included in the study.

According to the literature, mood disorders have long been associated with light and circadian rhythms [32]. One example is seasonal affective disorder, in which the mood oscillates between dysthymia during the short winter days and euthymia during the long days of summer. Circadian regulation concerns most systems believed to control the mood, including limbic brain regions, monoamine neurotransmitters, and the hypothalamic–pituitary–adrenal axis. Therefore, exposure to artificial light at night is believed to contribute to the prevalence of mood disorders [33]. A systematic review and meta-analysis showed a significant association between exposure to ALAN and an increased risk of depression. An increase of 1 nW/cm^2^/sr in outdoor ALAN corresponded to a 0.43% higher risk of depression [34].

There is little evidence to support a cause-and-effect relationship between eye diseases and circadian rhythms, although it is well known that people with blindness experience poorly entrained circadian rhythms, sleep disturbances, and a depressed mood. According to some studies, circadian disruption may be caused by inadequate exposure to daylight, by ophthalmic disease that reduces light signal transmission, or both [35]. Glaucoma affects light input to the circadian system and causes optic nerve dysfunction [36].

According to animal and human studies, melatonin demonstrates a significant hypotensive effect [37,38]. One night of moderate (100 lx) light exposure during sleep increases the nighttime heart rate, decreases heart rate variability, and increases next-morning insulin resistance when compared to sleep in a dimly lit (<3 lx) environment [39]. Working at night with artificial light is associated with an elevated blood pressure and hypertension [4]. In a cross-sectional study concerning 80,000 female nurses in China, working at least five nights per month increased the hypertension risk from 19% to 32% [40].

The results concerning quality of life measured with the WHOQOL-BREF are noteworthy. Residents of both villages were characterized by a reduced quality of life in the social domain in comparison to other areas studied. They were dissatisfied with their financial resources, which are the lowest in Poland, social support, and access to healthcare. Moreover, mean values in the environmental and physical domains of the WHOQOL-BREF were significantly higher among the inhabitants of the Dark Sky Park than among the respondents in the control town. Opportunities and participation in recreation and leisure, access to information, and factors describing the physical environment (noise, pollution, climate) are sample subscales in the environmental domain. The physical domain includes following subscales: activities of daily living, dependence on medications, rest and sleep, and ability to work. According to the design of this study, it is not possible to determine whether illumination or a better sleep in the Dark Sky Park has an impact on the overall health of residents. However, according to the WHOQOL-BREF scale, both relaxation and the ability to work, as well as environmental conditions in which they live, are superior among residents of the Dark Sky Park. At the same time, it is important to remember that residents of the DSP may be more aware of the impact of the environment on health.

According to Simons et al. (2018), results of the WHOQOL-BREF scale can depend on lighting [41]. In 2018, in the Netherlands, intensive care nurses were divided into two groups. Each group worked alternately for 3–4 days in patient rooms with dynamic lighting at levels up to 1700 lux during the daytime and for 3–4 days under control conditions (300 lux) [41]. Cognitive performance, symptoms of depression, fatigue, and well-being were assessed before and after each period via cognitive tests and the WHOQOL-BREF questionnaire. The subjective well-being scores of nurses were significantly lower after working in dynamic lighting. Moreover, nurses working in artificial light perceived that they had a significantly lower quality of life on the WHOQOL-BREF scale, particularly in the psychological and environmental domains [41].

ALAN leads to unnecessary energy losses. Currently, numerous countries are devoting considerable attention to this issue, with the aim of modifying the design of lighting devices and installations to reduce light pollution, save energy, and enhance the environmental safety of lighting. Many of the aforementioned changes are consequences of European Union (EU) policies aimed at reducing climate change [42]. Creating Dark Sky Parks is an element of these policies. The literature suggests that efforts to recognize the value of dark skies and support their conservation may have positive benefits in reducing skyglow on regional scales, as well as save energy [8].

Our findings should be interpreted in the context of this study’s design and limitations. Dark Sky Parks are often uninhabited areas used only for astronomical observations. Consequently, there are no papers concerning the health of people living in such locations and it is impossible to make direct comparisons with the results obtained in this study.

The strength of this study lies in its design and recruitment of respondents from two similar mountain villages characterized by different illuminances of outdoor lighting. This study is probably the first analysis of the health of people living in a Dark Sky Community. To date, no similar studies have been conducted to assess the incidence of selected health diseases, as well as the quality of life measured with a standardized questionnaire in a DSP. Hence there are also numerous limitations. First, potential bias may result from the fact that our study was based on a self-administered survey. In future studies, both sleep and the mood should be assessed using validated questionnaires. Second, the inhabitants were not asked about their work. The agricultural nature of both mountain villages and the distance 30 km from the nearest town virtually precluded the possibility of shift employment. Nevertheless, it is possible that some individuals were engaged in shift work that exposed them to light at night on a chronic or rotating basis. Moreover, it cannot be assumed that individuals were not exposed to artificial indoor light at home. They were asked about reducing the light coming through windows inside but nothing is known about the light generated at home. It also should be emphasized that the inhabitants of the Dark Sky Park might be more aware of the health consequences of exposure to ALAN due to events organized there. Most of the events are astronomical observations for astronomers but other activities cannot be ruled out. The medical history was not analyzed in this study. No scale was used to assess hypertension and other diseases. Finally, it should be noted that it is not possible to draw any conclusions about the cause and effect of the relationship found because of the cross-sectional nature of this study. This study only demonstrates the prevalence of certain diseases according to road lighting. The existence of various adverse health consequences associated with light pollution has been demonstrated in other studies.

## 4. Methods and Materials

As health effects of ALAN are related not only to the amount of light but also to the exposure conditions and characteristics of the light, it was decided to construct a ‘natural experiment’ to compare two geographically close and demographically similar communities, one with reduced roadway lighting.

Research material was collected among the inhabitants of two similar mountain villages in a Silesian Voivodeship (Poland), one of which has been a Dark Sky Park since 1997 and the first place in Poland designated to be the International Dark Sky Community. Both villages have about 2000 residents, and they are situated in two neighboring mountain valleys, 13 km apart. According to Statistics Poland 2024, the average gross wages and salaries in both villages were at the same lowest level and amounted to PLN 6831.33–8000.00 [43]. Houses in both villages are arranged along a road lit by luminaires. Roadway lighting in the DSP meets the standards of the International Dark-Sky Association (IDA). In 2011, street lighting in the DSP was modernized. The directionality of luminaires was improved, and the illuminance was reduced. In addition, at 11:00 p.m., the roadway lighting is completely turned off, allowing the implementation of astronomical observations. According to the Light Pollution Map, LP in the Dark Sky Park measured with the Bortle scale is 4.1 and in the control village 4.9 [44], and based on this, it was assumed that lighting in the DSP is lower than in the control town. Measurements of illuminance were not performed in this study.

This study was conducted in January 2022 according to international ethical standards. A self-administered questionnaire consisting of 16 single-choice questions was used. The questions concerned the duration and quality of sleep, sleeping pill administration, occurrence of selected diseases and disorders, and treatment. Furthermore, the Polish version of the WHOQOL-BREF standardized questionnaire was used for quality-of-life assessment [45]. The WHOQOL-BREF is a 26-item instrument consisting of four domains: physical health, psychological health, social relationships, and environmental health. It also contains quality of life and general health items. 

Participation in this study was voluntary and anonymous. General information about the purpose of this study and informed consent was placed on the first page of the questionnaire. Only respondents who read and signed the consent form could answer additional questions.

The sample was randomly selected. Invitations to participate in this study based on contact information (addresses only, without names or surnames) were randomly sent to a sample. Sample sizes for both villages were calculated using the sample size calculator (calculator.net). The number of adults was obtained separately for each village from Statistics Poland 2021 [43]. In the Dark Sky Park, there are 1649 residents, and 1387 of them are adults. In the control village, 2062 people are registered, of which 1586 are adults. Not all selected participants agreed to participate in this study. The survey was conducted among 272 respondents, 138 adult people from the DSP and 134 from the control town. As the sample confidence level was 95%, the maximum sampling error did not exceed 8%.

Only complete questionnaires were included in the statistical analysis. The Shapiro–Wilk W test was used to assess the normality of the distribution of variables. The characteristics of the respondents and other variables were compared between groups using the Mann–Whitney U-test or the Pearson chi-square test where appropriate. Cramér’s V was used to measure an effect size for the Pearson chi-square test of independence. For all analyses, the significance level was set at 0.05 and all calculations were performed using the software package Statistica v.13 (Statsoft, Cracow, Poland). Logistic regression models were employed to examine the relationship between variables.

## 5. Conclusions

The residents of both towns exhibited similar sleep patterns, but the inhabitants of the Dark Sky Park were statistically less likely to have difficulty falling asleep. They used sleeping pills, blackout curtains, and window blinds less frequently. Cardiovascular diseases, eye diseases, and mood disorders were declared less frequently among inhabitants living in an area with reduced illuminance. The quality of life in the physical and environmental domains was higher among the residents of the Dark Sky Park even though the salary level in both villages was equally low. People living in places with limited illuminance declare better health and a higher quality of life in the environmental and physical domains measured with the WHOQOL-BREF scale.

## Figures and Tables

**Table 1:** The characteristics of the study group (n = 272) (means ±SD, medians, IQR).

		Dark Sky Park (n = 138)	Control Town (n = 134)	Cramer’s V	*p*-Value
Age		46.99 ± 11.12	46.04 ± 11.82	0.550	0.459
Years of residence		44 (38–52)	40 (30–54)	0.458	0.502
Sex (%)	woman	59.42	68.42	0.096	<0.001
	man	40.58	31.58		
Education (%)	primary	4.35	5.97	0.056	<0.001
	vocational	34.06	38.06		
	secondary	44.93	35.82		
	higher	16.67	20.15		

**Table 2:** Sleep of respondents (n = 272).

		Percentage (%)		Cramer’s V	*p*-Value
		Dark Sky Park n = 138	Control Town n = 134		
Satisfaction with sleep	Very satisfied	21.01	8.21	0.376	<0.001
	Satisfied	53.62	31.34		
	Neither satisfied nor dissatisfied	14.49	23.88		
	Dissatisfied	7.25	29.10		
	Very dissatisfied	3.62	7.46		
The frequency of problems with sleeping	I have no problems with sleeping	58.70	49.25	0.17	0.04
	Just a few times in a month	30.43	27.61		
	Once a week	2.17	7.46		
	More than once a week	8.70	15.67		
Taking sleeping pills	Yes	4.35	17.16	0.21	0.00062
	No	95.65	82.84		
Sleeping habits	Curtains/blinds	26.81	62.69	0.41	0.0006
	Sleeping with the TV turned off	51.45	16.42		
	Sleeping with the TV turned on	20.29	17.16		
	Other, e.g., additional lighting turned on	1.45	3.73		

**Table 3:** Self-reported presence of depressed mood (n = 272).

		Percentage [%]		Cramer’s V	*p*-Value
		Dark Sky Park	Control Town		
The frequency of negative feelings, such as depression, anxiety	Very often	5.07	10.45	0.201	0.01
	Often	8.70	12.69		
	Rarely	32.61	42.54		
	Never	53.62	34.33		
Using the help of a specialist (psychologist, psychiatrist)	Yes	10.14	20.9	0.148	0.015
	No	89.86	79.1		

**Table 4:** Number of cases of selected diseases in the group of respondents according to their self-assessment (n = 272).

Selected Diseases	Percentage (%)		Cramer’s V	*p*-Value
	Dark Sky Park n = 138	Control Town n = 134		
Cancers	3.62	8.21	0.097	0.108
Diseases of the digestive system (e.g., peptic ulcer disease)	5.80	11.19	0.096	0.109
Heart disease (e.g., myocardial infarction)	7.25	9.70	0.044	0.46
Other cardiovascular diseases (e.g., hypertension)	21.74	33.58	0.132	0.028
Eye diseases (e.g., glaucoma)	1.45	8.21	0.158	0.008
Mood changes (e.g., depression, neurosis)	11.59	21.64	0.13	0.025
Eating disorders (e.g., bulimia, anorexia)	3.62	4.48	0.021	0.72

**Table 5:** Comparison of the results obtained in the WHOQOL-BREF test among the residents.

Domains of WHOQOL-BREF Questionnaire	Dark Sky Park n = 138		Control Town n = 134		*p*-Value
	Mean Value ± SD	Range (Min–Max)	Mean Value ± SD	Range (Min–Max)	
Quality of life	3.68 ± 0.74	1–5	3.64 ± 0.84	1–5	0.72
General health	3.93 ± 0.73	1–5	3.74 ± 0.82	1–5	0.06
Physical health	21.83 ± 3.29	11–32	21.04 ± 3.24	10–32	0.04
Psychological health	20.99 ± 2.82	10–27	20.57 ± 3.34	9–27	0.37
Social relationships	11.15 ± 2.65	3–15	11.14 ± 2.18	3–15	0.54
Environmental health	27.74 ± 3.42	14–40	26.29 ± 5.23	12–40	0.01

## Data Availability

The datasets used and/or analyzed during the current study are available from the corresponding author on reasonable request.
